# Bees' Honey Protects the Liver of Male Rats against Melamine Toxicity

**DOI:** 10.1155/2013/786051

**Published:** 2013-07-21

**Authors:** Haddad A. El Rabey, Madeha N. Al-Seeni, Suad M. Al-Solamy

**Affiliations:** ^1^Biochemistry Department, Faculty of Science, King Abdulaziz University, Jeddah, Saudi Arabia; ^2^Bioinformatics Department, Genetic Engineering and Biotechnology Research Institute, Minufiya University, P.O. Box 79, Sadat City, Egypt

## Abstract

The protective effect of natural bees' honey to the liver of male albino rats against melamine toxicity was studied. Melamine supplementation at a dose of 20000 ppm in the diet for 28 days induced adverse effects on the liver, decreased serum total protein and increased liver enzyme: alanine aminotransferase, aspartate aminotransferase, and alkaline phosphatase. Histological changes of the melamine supplemented group showed necrosis in the hepatic tissues around the central veins of the liver and precipitation of melamine crystals. Treating the male albino rats (that were presupplemented regularly with 20000 ppm melamine) with natural bees' honey at a dose of 2.5 g/kg body weight for 28 days improved both liver functions and increased serum protein. In addition, a positive impact on the shape of the cells after treatment with honey compared to the positive melamine supplemented group was observed. In conclusion, the results of this study revealed that the use of natural bees' honey has the ability to protect the liver of rats against the toxic effects of melamine.

## 1. Introduction

Melamine (2,4,6-triamino-1,3,5-triazine, 1,3,5-triazine-2,4,6-triamine, 2,4,6-triamino-s-triazine, melamine amide or cyanuric triamide) is a nitrogen heterocyclic triazine compound, referred to as triamines or protein essence [1]. Melamine contains 66% nitrogen by mass, so it is sometimes illegally added to food products in order to increase the apparent protein content that has recently become a serious concern [1, 2]. Standard tests such as the Kjeldahl and Dumas tests estimate protein levels by measuring the nitrogen content, so they can be misled by adding nitrogen-rich compounds such as melamine [3]. The illegal use of melamine as a food ingredient has led to many poisoning incidents of cats and dogs in the United States, as well as renal function failure of Chinese infants [4, 5]. 

The toxicity of melamine alone is very low, and greater than 90% of the ingested melamine is eliminated within 24 h in an animal experiment [6]. In spite of this lower toxicity of melamine, most animal studies showed effects on health following subacute or chronic melamine exposure [2, 6, 7]. Data of serum chemistry after one month of melamine-contaminated artificial food indicated severe renal and mild hepatic failure [8, 9]. Hau et al. [10] reported that more than 90% of ingested melamine is excreted within 24 h, the half-life of melamine excretion in animal studies ranged from 2.7 to 4.04 h, and the levels of melamine in blood, liver, or plasma are similar.

The Council of Agriculture in Taiwan ordered an emergency recall of the suspected pet food, and a surveillance committee was constituted to investigate [9]. Melamine contamination of baby milk-based products was detected in China [11]. In general, approximately 300,000 people were affected, more than 52,000 were hospitalized, and six died due to global marketing of melamine-contaminated products in almost 70 countries, including the United States [10, 12, 13].

 Gas chromatography-mass spectrometry (GC-MS) and high-performance liquid chromatography (HPLC) combined with ultraviolet (UV) or mass spectrometry (MS) detector are the most common methods used to detect melamine in foods and feedstuff [1, 14–16]. 

Honey is a sweet food made by bees using nectar from flowers. It is composed of a complex mixture of carbohydrates, proteins, enzymes (invertase, glucose oxidase, catalase, and phosphatases), amino and organic acids (gluconic acid, acetic acid, etc.), lipids, vitamins (ascorbic acid, niacin, pyridoxine, etc.), volatile chemicals, phenolic acids, flavonoids, and carotenoid-like substances and minerals which may function as antioxidants [17]. The composition of honey depends on the plant species visited by the honeybees and the environmental, processing, and storage conditions [18, 19]. The maillard reactions between amino groups of amino acids/proteins and reducing sugars lead to formation of a-dicarbonyl compounds such as glyoxal and methylglyoxal [20]. Honey is a natural antioxidant which may contain flavonoids, ascorbic acid, tocopherols, catalase, and phenolic compounds all of which work together to provide a synergistic antioxidant effect, scavenging and eliminating free radicals [21]. Its protective role against the kidney dysfunctions induced by sodium nitrite, a known food additives, hepatoprotective, hypoglycemic, reproductive, antihypertensive, and of course antioxidant effects has also been reported [22, 23]. 

The current study aimed to test the protecting effect of the natural bees' honey to the liver of male albino rat against melamine toxicity. 

## 2. Materials and Methods

All animal experiments were carried out under protocols approved by the Institutional Animal House of the University of King Abdulaziz, Jeddah, Saudi Arabia. 

### 2.1. Animals

Male albino rats (*Rattus rattus*) weighing about 180–200 g were obtained from King Fahd Center for Medical Research. 

### 2.2. Diets

Conventional animal basal diet was obtained from a Grain Silos & Flour Mills in Jeddah. This diet contains the following: crude protein (20.00%), crude fat (4.00%), crude fiber (3.50%), ash (6.00%), salt (0.50%), calcium (1.00%), phosphorus (0.60%), vitamin A (20,0 IU/g), vitamin D (2.20 IU/g), vitamin E (70.00 IU/g), and energy ME K Cal/kg (2850.00). Trace elements (cobalt, copper, iodine, iron, manganeses, selenium, and zinc) were added. Basal diet food was stored in a dry place out of direct sunlight.

Melamine (99%) was purchased from Sigma-Aldrich cat. no. M2659-5G.

Sidr bees' honey (from flowers of *Ziziphus spina-christi* trees, from Hadramout, Yemen) was purchased from a specialized shop in Jeddah, Saudi Arabia.

### 2.3. Experimental Design

Rats were held for approximately two weeks before the study began for acclimatization and then housed five per polycarbonate cages. Cages, bedding, and glass water bottles (equipped with stainless steel sipper tubes) were replaced twice per week. Test diets, control diets, and tap water are available *ad libitum.* Stainless steel feed containers were changed once per week. 

Forty rats were distributed into four groups each consists of 10 rats as follows.

1. Rats of the first group (G1) were fed diets containing normal basic diet served as untreated control group.
2. Rats of the second group (G2) were fed diets containing 20,000 ppm melamine to induce toxicity [24].
3. Rats of the third group (G3) received daily 2.5 g/Kg body weight of a 25% natural bees' honey in an aqueous solution by gastric tube [25] for 28 days.
4. Rats of the fourth group (G4) received the same dose of melamine as in the second group together with natural honey dose as in the third group for 28 days.

### 2.4. Biochemical Blood Tests

At the end of each specified period (28 days), rats were anesthetized using diethyl ether, and then, blood samples were collected from the orbital sinus [26]. Blood was collected in EDETA tube for CBC analysis and in plain tubes for chemistry analyses. Serum was obtained by centrifuging the blood samples at 1000 rpm for 10 min at room temperature and then stored at −20°C until analysis was performed.

Complete blood count (CBC) tests; hemoglobin (Hb), hematocrit (HCT), mean corpuscular hemoglobin (MCH), mean corpuscular hemoglobin concentration (MCHC), mean corpuscular volume (MCV), platelet count (PLT), red blood cell (RBC), and white blood cell (WBC) were measured using human reagent, Cell Dyne 17000, blood analysis system, Diagnostics Abbott, China, according to the instruction of the supplier.

Serum protein and albumin were measured using human reagent, Cobas—C111, fully automated clinical analyzers, Diagnostics Roche, India, according to the instruction of the supplier.

Serum liver enzymes, aspartate aminotransferase (AST), alanine aminotransferase (ALT), and alkaline phosphatase (ALP), were measured using human reagent, Cobas—C111, fully automated clinical analyzers, Diagnostics Roche, India, according to the instruction of the supplier.

### 2.5. Weight Gain, Body Weight Gain Ratio (BWG%), and Food Efficiency Ratio (FER)

Food intake and body weight per cage were recorded once per week. The mean body weight of each group was calculated by dividing the total weight of all surviving animals by the number of surviving animals in the group. Weight gain (g), body weight gain ratio (BWG%), and food efficiency ratio (FER) were calculated as follows:

- weight gain = final weight (g) − initial weight (g),
- BWG% = final weight − initial weight/initial weight × 100, and
- FER = weight (g)/food intake (g).

### 2.6. Histopathology

Animals were then scarified by cervical dislocation. The abdomen was opened, and the liver was rapidly dissected out, washed in sterile saline, and kept in 10% formalin. For microscopic preparations, the liver was dehydrated in gradual ethanol (50–99%), cleared in xylene, and embedded in paraffin. Sections were prepared and then stained with hematoxylin and eosin dye for microscopic investigation [27]. The stained sections were examined and photographed using a light microscope. 

### 2.7. Statistical Analysis

Data were analyzed using the Statistical Program for Sociology Scientists (SPSS) Statistics Version 17.0 for computing the mean values, the standard errors (SE), and the test of significance (*t*-test). Histograms were plotted using Excel program.

## 3. Results

### 3.1. CBC

The effect of honey treatment on the CBC in rats supplemented with melamine for 28 days is illustrated in Table 1. The mean values of hemoglobin in the positive control (G2, rats supplemented with 20000 ppm melamine) were significantly lower (*P* < 0.01) than those of the negative control. In G3 (rats supplemented with honey), the mean values of hemoglobin were significantly higher (*P* < 0.001) than those of the negative control, while the mean values of hemoglobin in G4 (rats supplemented with melamine and treated with honey) were nonsignificantly lower than those of the negative control and higher than those of the positive control.

The mean values of hematocrit in the positive control were significantly lower (*P* < 0.01) than those of the negative control. In G3, the mean values of HCT were significantly higher (*P* < 0.001) than those of the negative control, whereas the mean values of HCT in G4 were nonsignificantly lower than those of the negative control and higher than those of the positive control.

The mean values of RBC in the positive control were significantly lower (*P* < 0.05) than those of the negative control. In G3, the mean values of RBC were significantly higher (*P* < 0.001) than those of the negative control, while the mean values of RBC in G4 were nonsignificantly lower than those of the negative control and higher than those of the positive control. 

The mean values of MCV in the positive control were significantly lower (*P* < 0.05) than those of the negative control. In G3, the mean values of MCV were non significantly higher than those of the negative control. While the mean values of MCV in G4 were non significantly lower than those of the negative control and higher than those of the positive control. 

The mean values of MCH in the positive control were significantly lower (*P* < 0.01) than those of the negative control. In G3, the mean values of MCH were significantly higher (*P* < 0.01) than those of the negative control, while the mean values of MCH in G4 were non significantly lower than those of the negative control and higher than those of the positive control. 

The mean values of MCHC in the positive control were nonsignificantly lower than those of the negative control. In G3, the mean values of MCHC were significantly higher (*P* < 0.01) than those of the negative control. While the mean values of MCHC in G4 were significantly higher (*P* < 0.001) than those of the negative and positive controls. 

 The mean values of WBC in the positive control were significantly higher (*P* < 0.001) than those of the negative control. In G3, the mean values of WBC were significantly higher (*P* < 0.01) than those of the negative control, while the mean values of WBC in G4 were significantly higher (*P* < 0.01) than those of the negative control and lower than those of the positive control. 

 The mean values of platelet count in G2 and G3 were nonsignificantly higher than those of the negative control, while the mean values of PLT in G4 were non significantly lower than those of the negative control and positive control. 

### 3.2. Serum Proteins

Table 2 shows the effect of honey treatment for 28 days on serum proteins in rats supplemented with melamine. The mean values of total protein in the positive control were nonsignificantly lower than those of the negative control, whereas in G3, the mean values of total protein were nonsignificantly higher than those of the negative control. The mean values of total protein in G4 were nonsignificantly higher than those of the negative control and the positive control. 

The mean values of albumin in G2 and G3 were nonsignificantly lower than those of the negative control, while the mean values of albumin in G4 were lower than those of the negative control and higher than those of the positive control. 

### 3.3. AST, ALT, and ALP

**Figure 1:**
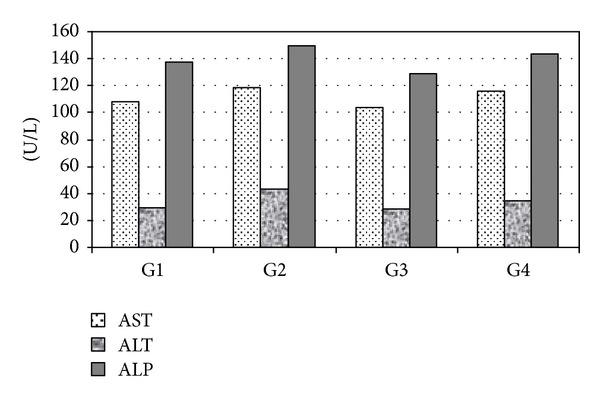
Effect of honey treatment on serum liver enzyme in rats supplemented with melamine for 28 days.

Figure 1 shows the effect of honey treatment for 28 days on liver enzymes in rats presupplemented with melamine. The mean values of AST in the positive control were nonsignificantly higher than those of the negative control (118.20 ± 9.78 and 107.80 ± 2.65 U/L, resp.). In G3, the mean values of AST were nonsignificantly lower than that of the negative control (103.66 ± 4.31 and 107.80 ± 2.65 U/L, resp.), while the mean values of AST in G4 were nonsignificantly higher than those of the negative control and lower than those of the positive control (115.46 ± 2.78, 107.80 ± 2.65, and 118.20 ± 9.78 U/L, resp.). 

The mean values of ALT in the positive control were significantly higher (*P* < 0.01) than those of the negative control (43.40 ± 3.14 and 29.75 ± 1.98 U/L, resp.). In G3, the mean values of ALT were significantly lower (*P* < 0.01) than those of the negative control (28.32 ± 2.15 and 29.75 ± 1.98 U/L, resp.), while the mean values of ALT in G4 were significantly higher (*P* < 0.01) than those of the negative control and lower than those of the positive control (35.00 ± 0.83, 29.75 ± 1.98, and 43.40 ± 3.14 U/L, resp.). 

The mean values of ALP in the positive control were significantly higher (*P* < 0.05) than those of the negative control (149.75 ± 10.01 and 137.60 ± 9.42 U/L, resp.). In G3, the mean values of ALP were significantly lower (*P* < 0.05) than those of the negative control (128.75 ± 4.61 and 137.60 ± 9.42 U/L, resp.), while the mean values of ALP in G4 were nonsignificantly higher than those of the negative control and lower than that of the positive control (143.50 ± 10.51, 137.60 ± 9.42, and 149.75 ± 10.01 U/L, resp.). 

### 3.4. Food Intake, Weight Gain, BWG%, and FER

The effect of melamine toxicity and honey treatment for 28 days on food intake, weight gain, BWG%, and FER in rats under study is shown in Table 3. The mean values of food intake (FI) were increased in the positive control (G2) compared to those of the negative control group. Meanwhile, G4 showed a decreased in food intake compared to the positive control group (G2).

As shown in Table 3, weight gain, body weight gain ratio, and food efficiency ratio were decreased in positive control compared to the negative control group, while G4 showed a decrease in weight gain and increase in body weight gain ratio and food efficiency ratio compared with the positive control group.

**Figure 2:**
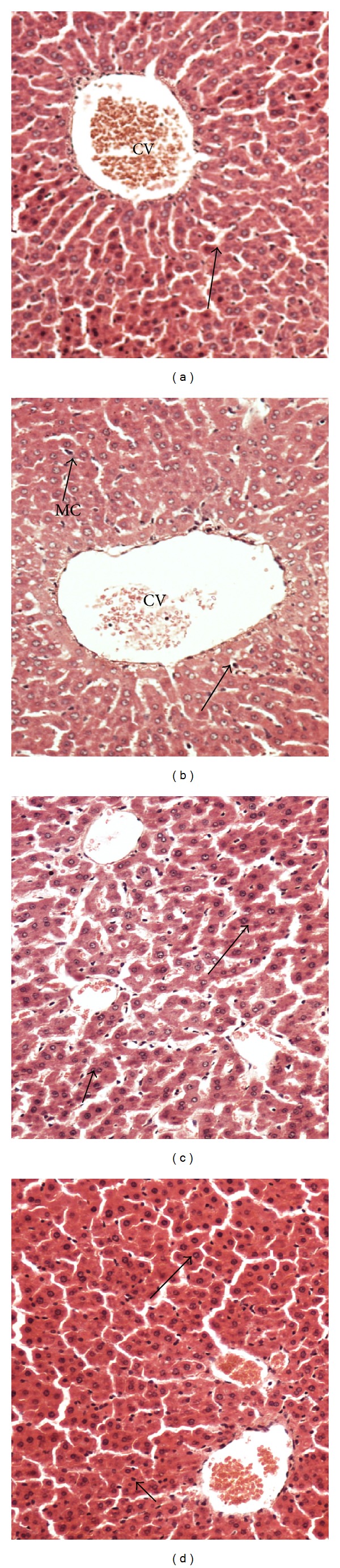
(a) Normal hepatic tissues showing hepatic strands of cells around the central vein (CV) leaving blood sinusoids (arrow). (b) Hepatic tissues pretreated with melamine show highly degree of necrosis hepatic tissues around the central veins (short arrow), highly degenerated hepatic tissues, and accumulation of granules in hepatic tissues (long arrow). Note dramatic pathological changes and accumulation of hepatic granules in most tissue and melamine crystals (MC). (c) Hepatic tissue of rats treated with honey showing normal hepatic tissues with normal hepatic strands (arrows). (d) Hepatic tissues supplemented with melamine and treated with honey show slightly necrotic hepatic tissues (short arrow) with normal hepatic strands (long arrow). ×200 (H&E stains).

Figure 2(a) shows the normal hepatic tissues of the control group with hepatic strands of cells around the central vein leaving blood sinusoids, whereas the liver tissue of (G2) which was regularly supplemented with 20000 ppm melamine (Figure 2(b)) shows massive fatty changes, necrosis, and broad infiltration of the lymphocytes comparable to those of the control group. The histological architecture of liver sections also showed more or less abnormal patterns, with a mild degree of necrosis and slightly lymphocyte infiltration, almost comparable to those of the control group with well-preserved cytoplasm and prominent nucleus that contains clusters of round blue melamine crystals. Also the hepatic tissue treated with honey showed normal hepatic tissues with normal hepatic strands (Figure 2(c)). The histological examination of liver sections supplemented with 20000 ppm melamine and honey-treated animals (Figure 2(d)) showed slightly degree of necrosis and clear accumulation of hepatic strands.

## 4. Discussion

The present investigation has been focused on testing the protective effects of oral administration of honey on melamine-induced liver dysfunction. In the present study, rats treated with melamine showed a significant decrease in hemoglobin, hematocrit, mean corpuscular hemoglobin concentration, and red blood cells after 28 days compared to the control. In addition, the mean corpuscular hemoglobin (MCH) and mean corpuscular volume (MCV) values were decreased after the consumption of melamine in diet of rats for 28 days. These findings agree with other investigations that showed significant decline in the mean cell volume and mean cell hemoglobin values in rats exposed to pet food contaminated with melamine and cyanuric acid [9]. The observed elevation in white blood cell after melamine supplementation for 28 days is also in agreement with the above-mentioned study [9]. 

Treating rats with honey during melamine supplementation for 28 days increased the mean values of serum hemoglobin and red blood cell, compared to that of the negative control. This result disagree with another study that showed that honey administration tends to have stabilizing effects as there were no effects on hemoglobin, red blood cell counts at 20% (v/v)-treated group corroborate the honey as an antianemic and immunostimulant agent [28]. Also, the data obtained from the present study showed an increase in white blood cell and mean corpuscular hemoglobin values after oral administration of bees' honey for the experiment time span. This observation is concordant with [28], who recorded an increase in mean corpuscular hemoglobin and white blood cell counts at 20% (v/v) treated group in male rats. This might be due to the protective effect of honey which contains moisture, sugars such as glucose and fructose, enzymes such as catalase and glutathione reductase, trace essential elements such as iron, copper, zinc, and calcium, vitamins such as vitamin A, C, and E, and some flavonoids and phenolic acids [29–31]. In addition, administration of honey has significantly attenuated the detrimental effect of poisonous materials on different organs of the rat [28]. 

 The increase in the mean values of platelet count after the oral supplementation of bees' honey in the current study is concordant with other investigations [32], which showed an increase in platelet count and disagree with the decrease in MCV and MCHC in rats that were not treated with bees' honey [32].

The decrease in serum total protein and albumin mean values of male rats under study after melamine supplementation in diet for 28 days is concordant with other results [9], which revealed a significant decline in albumin levels to pet food contaminated with melamine and cyanuric acid. 

The noticeable increase in serum contents of total protein and albumin with decrease in the activities of serum liver enzymes detected after oral administration of bees' honey to rats that received melamine is consistent with other investigations [33], which indicated a marked hepatoprotection induced by bees' honey.

On the other hand, melamine toxicity increased the levels of AST, ALT, and ALP in the melamine-treated group, compared to the negative control group. This finding agrees with other investigations [8], which showed an outbreak of pet food-associated severe renal failure and mild hepatic damage. The result of ALP disagrees with another study that showed that decrease of alkaline phosphatase in cats consumed melamine and cyanuric acid contaminated food [6].

Body weight gain was decreased in rats treated with melamine for 28 days compared to that of the negative control. This result is in agreement with other investigations [34, 35]. The decrease in body weight gain after oral administration of bees' honey is also consistent with other investigations [36, 37], suggesting that some other bioactivity of the honey plays a part such as insulinmimetic effects of the hydrogen peroxide produced by the honey [36], but it is unclear if hydrogen peroxide reaches sufficient levels in vivo to elicit such response. Alternatively, differences in weight gain may be a result of the high antioxidant content of honey such as the effect of green tea on rats [38]. The decrease of BWG% and FER as a result of melamine supplementation is also consistent with previous investigations [35]. In contrast, BWG% and FER were elevated after treatment with honey.

 Liver sections of rats supplemented with 20000 ppm melamine showed degenerated hepatic tissues, necrosis, and changes in massive fatty and broad infiltration of the lymphocytes. These results may indicate an increase in the release of the liver enzyme in the blood stream [39]. The concurrent administration of honey and melamine showed improvement in renal and hepatic tissues. These results are consistent with those reported in the previous literature [40].

 In conclusion, this work revealed the protective effect of honey to the liver of rats against melamine toxicity. This was concluded from the improvement in all biochemical tests and histopathological signs compared with the melamine-supplemented rats. This protective effect of honey may be attributed to the biologically active compounds such as vitamins, flavonoids, and antioxidants that work together to scavenge free radicals. Therefore, bees' honey can be used to protect animals and humans against the adverse effects of melamine toxicity.

## Figures and Tables

**Table 1:** Effect of honey treatment on CBC in rats supplemented with melamine for 28 days.

Test	Statistics	G1	G2	G3	G4
Hb (g/dL)	Mean ± SE	17.40 ± 0.39	15.54 ± 0.27	17.77 ± 0.29	15.67 ± 0.14
	*t*-test		3.04**	4.80***	−0.70 N.S
HCT%	Mean ± SE	52.70 ± 0.23	46.26 ± 1.54	53.00 ± 0.47	46.62 ± 0.66
	*t*-test		3.87**	5.29***	−0.20 N.S
RBC mil/cmm	Mean ± SE	9.66 ± 0.08	8.79 ± 0.31	10.17 ± 0.13	9.31 ± 0.06
	*t*-test		2.44*	4.73***	−1.85 N.S
MCV (fL)	Mean ± SE	54.40 ± 0.71	50.30 ± 1.39	54.50 ± 0.91	50.46 ± 0.12
	*t*-test		2.43*	2.05 N.S	−0.11 N.S
MCH (pg)	Mean ± SE	17.36 ± 0.22	16.16 ± 0.31	18.22 ± 0.33	16.47 ± 0.17
	*t*-test		2.87**	4.31**	−1.50 N.S
MCHC (g/dL)	Mean ± SE	31.88 ± 0.15	31.36 ± 0.22	32.87 ± 0.37	32.58 ± 0.04
	*t*-test		1.98 N.S	3.06**	−6.31***
WBC/cmm	Mean ± SE	7.800 ± 0.68	13.80 ± 0.81	8.47 ± 0.60	10.72 ± 1.00
	*t*-test		−11.01***	−4.12**	3.15**
PLT/cmm	Mean ± SE	466.43 ± 208.02	575.34 ± 157.51	482.00 ± 215.18	308.80 ± 168.47
	*t*-test		−0.52 N.S	0.31 N.S	1.25 N.S

*Significant at 5% (*P* < 0.05), **highly significant at 1% (*P* < 0.01), ***very highly significant at 0.1% (*P* < 0.001), and N.S: nonsignificant.

**Table 2:** Effect of honey treatment on serum protein in rats supplemented with melamine for 28 days.

Test	Statistics	G1	G2	G3	G4
Total protein g/dL	Mean ± SE	6.04 ± 0.38	5.79 ± 0.17	6.34 ± 0.10	6.28 ± 0.16
	*t*-test		0.19 N.S	1.56 N.S	1.00 N.S
Albumin g/dL	Mean ± SE	4.25 ± 0.18	4.10 ± 0.130	4.14 ± 0.11	4.20 ± 0.06
	*t*-test		1.53 N.S	0.24 N.S	0.72 N.S

N.S: nonsignificant.

**Table 3:** Effect of honey treatment on food intake, weight gain, BWG%, and FER in rats supplemented with melamine for 28 days.

Test	Statistics	G1	G2	G3	G4
Food intake g/week	Mean ± SE	1137.20 ± 51.34	1406.30 ± 192.99	1057.30 ± 9.27	1055.90 ± 62.69
	*t*-test		−1.12 N.S	−1.73 N.S	−1.53 N.S
Weight gain (g)	Mean ± SE	110.20 ± 16.55	92.48 ± 9.74	90.16 ± 14.07	108.72 ± 26.24
	*t*-test		1.05 N.S	−0.15 N.S	0.54 N.S
BWG%	Mean ± SE	54.66 ± 9.13	52.72 ± 6.85	56.09 ± 4.44	64.93 ± 20.50
	*t*-test		0.19 N.S	0.91 N.S	0.55 N.S
FER	Mean ± SE	0.119 ± 0.017	0.081 ± 0.008	0.105 ± 0.017	0.128 ± 0.031
	*t*-test		2.14*	1.40 N.S	1.36 N.S

*Significant at 5% (*P* < 0.05) and N.S: nonsignificant.
